# Semiconducting Nanoparticles: Single Entity Electrochemistry and Photoelectrochemistry

**DOI:** 10.3389/fchem.2021.688320

**Published:** 2021-06-02

**Authors:** S. Mathuri, Yuanhang Zhu, Mudaliar Mahesh Margoni, Xiuting Li

**Affiliations:** ^1^ Institute for Advanced Study, Shenzhen University, Shenzhen, China; ^2^ Institute of Microscale Optoelectronics, Shenzhen University, Shenzhen, China; ^3^ Crystal Growth and Thin Film Laboratory, Department of Physics and Nanotechnology, SRM Institute of Science and Technology, Kancheepuram, India

**Keywords:** semiconducting nanoparticles, single entity, (photo) electrochemistry, metal oxides, quantum dots

## Abstract

Semiconducting nanoparticles (SC NPs) play vital roles in several emerging technological applications including optoelectronic devices, sensors and catalysts. Recent research focusing on the single entity electrochemistry and photoelectrochemistry of SC NPs is a fascinating field which has attained an increasing interest in recent years. The nano-impact method provides a new avenue of studying electron transfer processes at single particle level and enables the discoveries of intrinsic (photo) electrochemical activities of the SC NPs. Herein, we review the recent research work on the electrochemistry and photoelectrochemistry of single SC NPs *via* the nano-impact technique. The redox reactions and electrocatalysis of single metal oxide semiconductor (MOS) NPs and chalcogenide quantum dots (QDs) are first discussed. The photoelectrochemistry of single SC NPs such as TiO_2_ and ZnO NPs is then summarized. The key findings and challenges under each topic are highlighted and our perspectives on future research directions are provided.

## Introduction

Semiconductor nanomaterials have attracted increasing interest due to their excellent physical and chemical properties comparing with their bulk counterparts, such as continuous absorption bands, narrow and intensive emission spectra, high chemical and photo-bleaching stability, processability and surface functionality, which make them suitable candidates in single electron devices, sensors, imaging devices, solar cells, nano-electronics, optoelectronic devices and memory devices (Itoh et al., 1988; Wang and Herron, 1991; Bhargava et al., 1994; John and Singh, 1996; Singh and John, 1997). Electrochemical and photo-electrochemical studies of semiconductor nanomaterials are important for understanding the mechanism and kinetics of the relevant processes in their applications. Macroscopic (ensemble) measurements are still predominantly used to infer the underlying microscopic processes when investigating the (photo) electrochemistry of nanomaterials. However, this is not always straightforward or fully representative. A large population of nanoparticles may result in polydispersity, different particle orientations, the formation of the likely agglomerated and irregular “mat” associated with the dropcast technique and other phenomena that make the results difficult to interpret. A greater mechanistic understanding of the electrochemical and photo-electrochemical reactivity of nanomaterials requires the development of method for probing electron transfer events on single redox nanomaterials and individual catalytic entities. One of these emerging methods is the “nano-impact” approach (or particle-electrode impacts) which is realising an entirely fresh way of studying nanoparticles, their reactivity and catalytic properties, transforming the science of nano-chemistry (Cheng and Compton, 2014; Pumera, 2014; Rees, 2014; Sokolov et al., 2017). The phenomenon of “nano-impact” method, due to Brownian collisions of NPs with an electrode held at a suitable potential, enables NPs to be individually electro-reduced or oxidised, or mediate (catalyze) charge transfer processes which are not viable on the microelectrode itself under the conditions of study. This powerful electrochemical technique has found much strength in giving insights into the fundamental study of nanoparticles: not only the basic particle characterization (e.g. sizing, concentration, chemical identity, agglomeration/aggregation state, porosity) (Zhou et al., 2011; Stuart et al., 2012; Tschulik et al., 2014; Jiao et al., 2017; Li et al., 2019), but also in-depth understanding at single-particle levels for the mechanisms and dynamics of (photo) electrochemical processes of interest (Xiao and Bard, 2007; Bard et al., 2010; Fernando et al., 2013; Li et al., 2016; Ma et al., 2018; Peng et al., 2018).

The stochastic electrochemistry “nano-impact” was born since Lemay’s report in 2004, where the collision of single latex microspheres on (ultramicroelectrode) UMEs was discussed (Quinn et al., 2004). Research at early stage mainly include the works by Quinn et al., 2004 and Xiao and Bard, 2007 on insulating and metallic NPs. The motivation for studies of semiconducting (SC) NPs starts from the interest in investigating photoelectrochemical currents for energy conversion in SC nanostructures. Since then, single entity studies on the semiconducting materials such as metal oxides and some quantum dots materials have been performed (Sardesai et al., 2013; Tschulik, et al., 2013; Perera et al., 2015; Fernando et al., 2015; Shimizu et al., 2017; Alshalfouh et al., 2019; Karunathilake et al., 2020; Wang et al., 2021). The overarching goal of studying single SC (photo) electrochemistry is to understand the net (photo) electrochemistry catalytic process at single particle level and to establish their instinct activity-structure relationship. Moreover, the mechanistic investigation on (photo) electrochemistry of individual NPs will contribute to understand and ultimately control the charge transfer at nanoscale. Even though several reports and reviews have summarised single SC photo-electrochemistry (Peng et al., 2018; Barakoti et al. (2016); Ma et al., 2018; Alpuche-Aviles et al., 2019; Wang et al., 2019; Wang et al., 2020), their electrochemical redox behavior and electro-catalytical activities were not comprehensively included. Therefore, our distinct aim of the mini review is to facilitate insight into both the electrochemistry and photoelectrochemistry of single semiconducting nanoparticles. A summarized data collection containing the electrochemistry and photoelectrochemistry of single entity semiconducting materials *via* nano-impact approach is presented in Table 1.

**TABLE 1:** Literature on single entity electrochemistry and photoelectrochemistry of semiconducting nanoparticles based on the nano-impact method.

Semiconducting materials	Measurement methods^a^	Research topics	References
ZnO	LSV and CA (nano-impact)	Electrochemical reduction	Karunathilake et al. (2020)
ZnO	CV and CA (nano-impact)	Photoelectrochemistry for water oxidation	Ma et al. (2018)
ZnO	LSV and CA (nano-impact)	Electrochemical reduction	Perera et al. (2015)
TiO_2_/IrO_x_	CV and CA (nano-impact)	Photoelectrochemistry for water oxidation	Wang et al. (2020)
TiO_2_	CV and CA (nano-impact)	Photoelectrochemistry for oxidizing I^−^	Peng et al. (2018)
TiO_2_	CV and CA (nano-impact)	Surface-bound electrochemical reduction (Alizarin Red S)	Shimizu et al. (2017)
TiO_2_	CV and CA (nano-impact)	Photoelectrochemistry for oxidizing the dye N719	Barakoti et al. (2016)
TiO_2_	CV and CA (nano-impact)	Photoelectrochemistry for oxidizing MeOH	Fernando et al. (2015)
TiO_2_	CV and CA (nano-impact)	Photoelectrochemistry for oxidizing MeOH	Fernando et al. (2013)
CuO	CV and CA (nano-impact)	Electrochemical reduction	Zampardi et al. (2018)
IrOx	CV and CA (nano-impact)	Electrocatalysis toward H_2_O_2_ oxidation	Zhou et al. (2017)
IrO_X_	CV and CA (nano-impact)	Electrocatalysis toward OER	Kwon et al. (2010)
CeO_2_	CV and CA (nano-impact)	Surface-bound electrochemical reduction (As^3+^)	Karimi et al. (2019)
CeO_2_	CV and CA (nano-impact)	Surface-bound electrochemical reduction (O_2_^−^)	Sardesai et al. (2013)
Fe_2_O_3_	CV and CA (nano-impact)	Electrochemical reduction	Shimizu et al. (2016a)
Fe_2_O_3_	CA (nano-impact)	Electrochemical reduction of agglomerates	Shimizu et al. (2016b)
Fe_3_O_4_	CA (nano-impact)	Electrochemical reduction	Tschulik et al. (2014)
Fe_3_O_4_	CV and CA (nano-impact)	Electrochemical redox behavior	Tschulik et al. (2013)
Co_3_O_4_	CV and CA (nano-impact)	Electrocatalysis toward water oxidation	Xie et al. (2020)
CoFe_2_O_4_	CV and CA (nano-impact)	Electrocatalysis toward OER	El Arrassi et al. (2019)
Al_2_O_3_	CV and CA (nano-impact)	Surface-bound electrochemical oxidation (catechol, anthraquinone, chloranil and poly(vinylferrocene))	Lin and Compton (2017)
Al_2_O_3_	CV and CA (nano-impact)	Surface-bound electrochemical oxidation (catechol)	Lin and Compton (2015)
CdSe QDs	CV and CA (nano-impact)	Electrochemical oxidation	Alshalfouh et al. (2019)
MoS_2_ QDs	CV and CA (nano-impact)	Electrocatalysis toward HER	Wang et al. (2021)

a Note that LSV is linear scan voltammetry, CV represents cyclic voltammetry and CA is chronoamperometry.

## Single Entity Electrochemistry of Semiconducting Nanoparticles

### Single Entity Electrochemistry of Metal Oxides Nanoparticles

As an important type of semiconducting materials, metal oxide semiconductors (MOS) attain great attention due to its morphological versatility, chemical stability, physicochemical interfacial properties and their ability to combine in composite structures (Santos et al., 2016; Liu et al., 2017; Dong et al., 2017; Zhang et al., 2020). The earliest electrochemical studies on MOS usually focus on the average behavior *via* ensemble measurements at a macroelectrode with a few milli-meters in diameter, some of which involve the simultaneous collision of the suspended colloids with the electrode surface (Dunn et al., 1981a; Dunn et al., 1981b; Kamat, 1985; Koelle et al., 1985; Gratzel and Frank, 1982) For example, polarography and voltammetry of aqueous SnO_2_ and TiO_2_ suspensions was investigated by Heyrovsky et al., in 1995 (Heyrovsky et al., 1995a; Heyrovsky et al., 1995b). Even though collision and adsorption of nanoparticles can take place on a macroelectrode, exceedingly high frequencies of collision and huge background signals associated with the large electrode area make it challenging to individually resolve the collision events. When a microelectrode is employed, both the collision frequency and baseline noise are greatly reduced, thereby resulting in clear resolution of single impact events. With the development of this nano-impact technique, understanding the property and activity of single MOS are attracting increasing interests. In recent years, the electrochemical behavior of single MOS including IrO_x_, Fe_2_O_3_, Fe_3_O_4_, CeO_2_, CuO, Co_3_O_4_, TiO_2_, ZnO and CoFe_2_O_4_ etc. has been investigated *via* nano-impact method (Sardesai et al., 2013; Tschulik and Compton, 2014; Shimizu et al., 2016a; Shimizu et al., 2016b; Zhou et al., 2017; Zampardi, et al., 2018; Peng et al., 2018; Xie et al., 2020; Karunathilake et al., 2020) The specific research topics mainly involve two aspects: the electrochemical redox behavior and electrocatalysis of single MOS, which is summarized and discussed in the following paragraphs respectively. For a direct type of nano-impact, the nanoparticles are usually fully oxidized (or reduced) so that the electrolysis of the nanoparticles is quantitative. The charge (Coulombs) obtained by integrating the current transients reflects (*via* Faraday’s first Law) the number of atoms (or molecules) in the nanoparticle thus giving its size. A large number of current spikes are easily and rapidly measured so giving a particle size distribution. The method is capable of sizing nanoparticles as small as 5 nm with suitably sensitive home-built but inexpensive equipment (Batchelor-McAuley et al., 2015). In addition to sizing nanoparticles the nano-impact technique is able to simultaneously measure their concentration *via* the frequency of the observed impacts (Stuart et al., 2012). Furthermore, the potentials at which the current spikes onset are clearly related to the chemical identity of the nanoparticles and recent work has shown that it is able to measure the states of agglomeration/aggregation of the particles (Tschulik et al., 2014) and the porosity of the particles (Jiao et al., 2017). Currently, the direct nano-impact studies on MOS mainly focus on the redox behavior of the MOS themselves Fe_3_O_4_ (Tschulik and Compton, 2014), Fe_2_O_3_ (Shimizu et al., 2016a; Shimizu et al., 2016b), ZnO (Perera et al., 2015; Karunathilake et al., 2020; Ma et al., 2018) and CuO (Zampardi et al., 2018) and the MOS surface-bound with electroactive species CeO_2_ (Karimi et al., 2019), TiO_2_ (Shimizu et al., 2017) and Al_2_O_3_ (Lin and Compton 2015, 2017).

**FIGURE 1:**
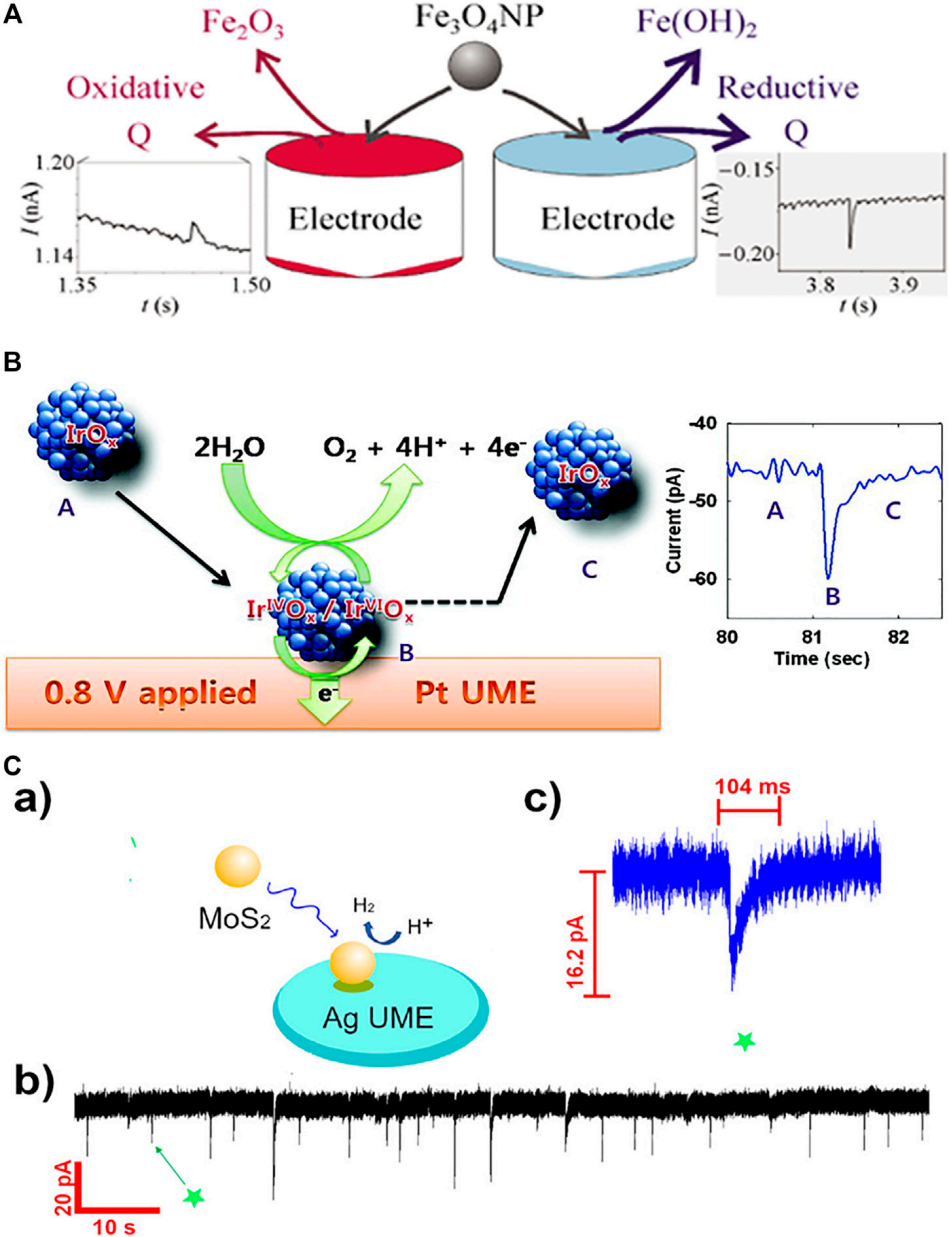
**(A)** The nano-impact measurements of single Fe_3_O_4_ magnetite nanoparticles using both anodic particle coulometry (APC) and cathodic particle coulometry (CPC) to independently get the size information of Fe_3_O_4_ nanoparticles (Tschulik et al., 2013). Copyright © 2013 Springer **(B)** Schematic illustration of single IrOx NP collision event and the current enhanced by electrocatalytic water oxidation (Kwon et al., 2010). Copyright © 2010 American Chemical Society **(C) (a)** Scheme of single MoS_2_ QD collision at the Ag UME surface; the reaction is switched on when the particle is in contact with the detection electrode. **(b)** Experimentally obtained current transient applied by Ag UME (diameter 10 μm) held at −700 mV vs SHE. **(c)** Representative current profile observed in a single QD collision event indicated in **(b).** Wang et al. (2021). Copyright © 2021 American Chemical Society.

Tschulik et al. conducted cathodic and anodic impact experiments for Fe_3_O_4_ nanoparticles and opened two independent routes to electrochemical sizing the particles (Figure 1A) (Tschulik et al., 2013). Furthermore, individual Fe_3_O_4_ NPs in the presence and absence of a magnetic field was investigated and a significant magnetic field-induced agglomeration of NPs is observed in a magnetic field. More interestingly, dissolution of Fe_3_O_4_ NPs is found to be strongly inhibited in a magnetic field; this is likely due to the magnetic field gradient force trapping the produced Fe^2+^ ions near the NP surface and hence hindering the mass-transport controlled NP dissolution (Tschulik et al., 2014). Shimizu et al. (2016b) achieved the electrochemical sizing for Fe_2_O_3_ NPs *via* conducting reductive impact experiments. It is found that the first electron transfer process is the rate determining step of the reductive dissolution of nanoparticles, and the interfacial proton concentration has strong effect on the overall process (Shimizu et al., 2016a; Shimizu et al., 2016b). A following work demonstrated the rapid and reversible agglomeration/dis-agglomeration process of Fe_2_O_3_ NPs and opens a new way of investigating the agglomeration equilibria of mineral nanoparticles in aquatic media (Shimizu et al., 2016a; Shimizu et al., 2016b; Shimizu et al., 2016). Perera et al. investigated the impact experiments of ZnO nanoparticles (NPs) and realised the determination of the redox potential for the ZnO NPs. It is observed that the formal potential is a strong function of NP size (1/r) since smaller NPs are less stable compared to the larger ones and hence relatively easier to be reduced. (Perera et al., 2015). Further understanding on the reduction kinetics and mass transport of ZnO single entities was reported by Karunathilake et al. (Karunathilake et al., 2020). Recently, to understand the environmental fate of CuO nanoparticles and further correctly assess their toxicity, Zampardi et al. employed the nano-impact method to investigate the electrochemical behavior of single copper oxide nanoparticles in the presence of anionic species (Cl^−^ and NO_3_^−^) commonly found in real water media. The potentials of *in-situ* detecting the nanoparticles in real world media is demonstrated (Zampardi et al., 2018).

In addition to the redox behavior of single MOS, the MOS absorbed with active species have also been investigated at single particle level. Based on the nano-impact method, Sardesai et al. studied the reduction of surface bound oxygen species on single CeO_2_ NPs and evaluated antioxidant activity of CeO_2_ NPs in a simple, rapid and inexpensive approach (Sardesai et al., 2013). Recently, the oxidation and reduction of As^3+^ loaded CeO_2_ NPs was reported by using collision electrochemistry and the highest spike frequency at pH 8 suggesting a maximum adsorption capacity. The As concentration in solution was determined by deriving from the measured charge and peak current, as well as the spike frequency (Karimi et al., 2019). In addition, the nano-impact is employed to probe the degree of cluster formation of Alizarin Red S modified rutile (TiO_2_) nanoparticles based on the reduction of the adsorbed dye molecules (Shimizu et al., 2017). Lin and Compton reported the quantification of the adsorbed catechol on single Al_2_O_3_ particles and the individual impact spikes resulting from the catechol oxidation was modeled to derive the charge diffusion coefficient across the particle surface (Lin and Compton, 2015). Adsorption of more redox active species including catechol, anthraquinone, chloranil and poly(vinylferrocene) on alumina particles was also investigated and their surface coverages and charge diffusion coefficients were determined respectively (Lin and Compton, 2017).

The investigation on redox behavior of single MOS and their absorption of redox active species discovers more accurate physiochemical properties for both MOS and the adsorbed redox species. As an alternative to the direct redox reaction of the nanoparticles themselves, mediated (indirect) electron transfer can also take place on the surface of impacting nanoparticles. When catalytically active nanoparticles stochastically collide with an inert microelectrode in a solution of redox molecules, transient current increases may be observed due to enhanced catalytic activity on nanoparticle surfaces. The current response of these collisions may adopt one of two general forms: a current spike or a current step, depending on a few complex factors such as the residence time of the impacting catalytic nanoparticles on the electrode surface and if the deactivation time of nanoparticle. If the desorption or deactivation of the nanoparticle is relatively slow compared to the experimental time, a “step on” in the current-time response is observed, otherwise a “spike” obtained. Some significant applications of the mediated electron transfer for MOS have been reported, examples cover IrO_x_, Fe_2_O_3_, Co_3_O_4_ and CoFe_2_O_4_ NPs. (Zhou et al., 2017; Kwon et al., 2010; Shimizu et al., 2016a,b; Xie et al., 2020; El Arrassi et al., 2019). The initial studies on the mediated impacts of MOS reported the enhanced current transients from the electrocatalysis of individual impacting IrO_x_ NPs toward oxygen evolution reaction (OER). A current decay rather than steady response was observed, and the obtained current is found to be highly sensitive to the material and surface state of the electrode used (Figure 1B) (Kwon et al., 2010). Coupled with microscopic investigation, the current transients from oxidation of hydrogen peroxide at single IrOx NP further discovers the origins of deactivation of catalytic NPs and the factors affecting the collision dynamics (Zhou et al., 2017). Recently Xie et al. reported the water oxidation on single Co_3_O_4_ nanoparticles, a mechanism involving hydrogen peroxide as the initial oxidation product of electron transfer and a following decomposition to form dioxygen was proposed. Single particle electrocatalysis points out the rate-determining step and the limiting kinetics of the reaction (Xie et al., 2020). El Arrassi et al. investigated the OER on single CoFe_2_O_4_ NPs and revealed that the current density at single nanoparticle researches as high as several kA·m^−2^. The analysis of the steady-state current further indicates that the electrocatalytic activity is limited by the diffusion of produced oxygen away from the particle, providing new insights into intrinsic activities of the nanocatalysts (El Arrassi et al., 2019).

Overall, the single entity electrochemistry of MOS covering the redox behavior and electrocatalysis of single MOS has been investigated *via* the nano-impact technique. The latter has shown the capability to reveal the fundamental physiochemical properties of nanoparticles (sizing, concentration, agglomeration/aggregation state), and to provide in-depth understanding the mechanisms and dynamics of electrochemical processes at nanoscale. However, more investigation on the redox behavior of single MOS in complicate aqueous media is required for finally realising the *in-situ* electrochemical determination and analysis of solution phase MOS in real world environment. Single entity electrocatalysis should be extended to more metal oxide based electro-catalysts for better understanding the underlying mechanism and kinetics of the important reactions.

### Single Entity Electrochemistry of Semiconducting Quantum Dots

In addition to the MOS, semiconducting quantum dots (QDs), especially chalcogenide QDs hold unique optical and electrical properties such as narrow emission absorption and high photo-stability, making them increasingly popular in recent years in the applications of optoelectronic devices, catalysis (Barakat et al., 2005), bio-labeling (Lin et al., 2007), lasers (Supran et al., 2013), sensors (Cho et al., 2015), LEDs (Yang et al., 2015) and photovoltaics (Yang et al., 2017). The research on single entity electrochemistry of QDs starts very recently with the report of CdSe/CdS QDs by Alshalfouh et al., 2019. Together with fluorescence correlation spectroscopy (FCS), electrochemical impact measurements was carried out to understand the reactivity as well as dynamics of CdSe/CdS QDs at a Pt microelectrode surface. The latter was around 1 μm in diameter for matching the size of the optical observation volume. Cyclic voltammetry was used to investigate the oxidation of CdSe/CdS QDs with negatively charged shells. Significantly, it is found that the electro-oxidized QDs are still able to emit light although the emission lifetime decreases. According to this report, different from the widely reported metal NPs in single collision experiment, more collision events are likely required for a small QD to have a complete anodic decomposition (Alshalfouh et al., 2019). According to this report, multiple collision events are required for a QD to have a complete anodic decomposition. What’s more, the intrinsic electrocatalytic activity of single MoS_2_ quantum dots (QDs) toward HER is just reported by Wang et al. (Figure 1C) (Wang et al., 2021). The current responses obtained at silver ultramicroelectrodes (UMEs) and carbon UMEs were recorded respectively. Current “spikes” with much higher current intensity is observed at Ag UMEs while “steps” obtained at C UMEs, revealing the influence of the substrate-MoS_2_ interface on HER activity. Very few reports on electrochemistry of single QDs so far is likely due to the following reasons: the small size of the QDs (a few nanometers in diameter) increases the difficulties to be electrochemically detected with the current low-noise potentiostats; the semiconducting nature of the QDs make the electron transfer process more complicate than the metal nanoparticles; the surface chemistry of the QDs imposed uncertainties to the collision process with the electrode surface.

## Single Entity Photoelectrochemistry of Semiconducting Nanoparticles

**FIGURE 2:**
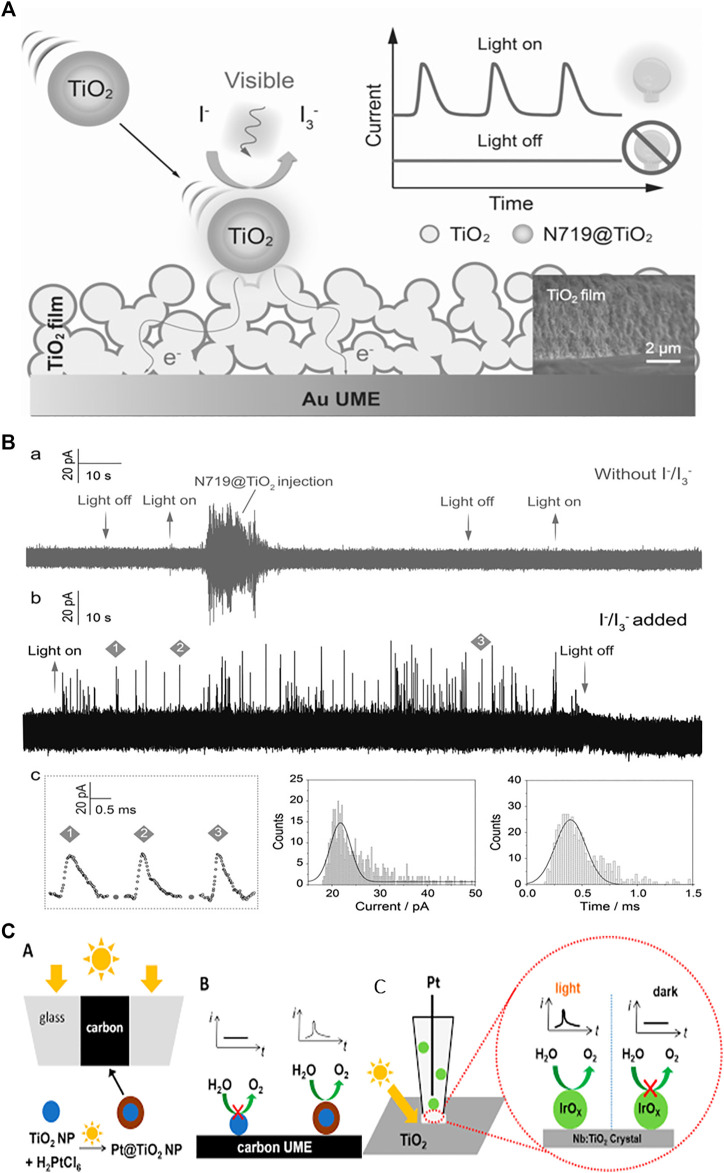
**(A)** Illustration of the photoelectrochemical behavior of a single N719@TiO_2_ nanoparticle during a collision with a TiO_2_@Au UME in the presence of I^−^/I^3−^ redox electrolyte under visible light, thus generating spike like current transients. **(B)** Amperometric current–time curves of individual N719@TiO_2_ nanoparticles at +600 mV vs. Ag/AgCl in the absence **(a)** and in the presence **(b)** of the I^−^/I_3_^−^ redox couple in the electrolyte solution under illumination with a Xe lamp (λ > 450 nm). **(c)** Expanded portions of the representative photocurrent traces. Histogram of the peak currents and duration time of individual transients. Peng et al. (2018). Copyright © 2018 John Wiley and Sons **(C)** Light controlled NP collisions: Schematic representation of **(A)** *in Situ* Photosynthesis of Pt@TiO_2_ NPs **(B)** their catalytically amplified collisions with the carbon UME and **(C)** Photoelectrochemically amplified collisions of IrO_x_ NPs with a microscopic portions of the Nb doped n-type TiO_2_ (110) Rutile single crystal surface facing the micropipette orifice Wang et al. (2020). Copyright © 2020 American Chemical Society.

Semiconductor photoelectrochemistry deals with solar energy conversion to electricity or chemical fuels and focuses on the photo-driven reactions at solid/liquid interfaces. To achieve the practical energy conversion applications, the fundamentals of SC nanomaterials such as charge carrier generation, separation, transport, and especially interfacial charge transfer at heterogeneous nanoscale interfaces need to be clarified. Single entity photoelectrochemistry of colloidal bared and sensitized TiO_2_, and ZnO NPs has been reported so far (Fernando et al., 2013; Fernando et al., 2015; Barakoti et al., 2016; Ma et al., 2018; Peng et al., 2018; Wang et al., 2020). Fernando et al. employed the nano-impact method to detect the stochastic photoelectrochemical currents of individual anatase TiO_2_ nanoparticles. It is reported that the current steps were not observed for NP suspension under illumination in MeCN while the steps appeared in MeOH. This is due to that NPs under illumination produce valence-band holes that oxidize MeOH. They have also investigated the photoelectrochemical behaviour of the NP with different diameter and reported that the collisions resulting in a current step could be smaller for the semiconductor NP than the metal NPs (Fernando et al., 2013). Furthermore, it has been demonstrated that the sensitivity of the detection can be improved by using a dye (Fernando et al., 2015; Barakoti et al., 2016; Shimizu et al., 2017; Ma et al., 2018; Peng et al., 2018). Specifically, Barakoti et al. (2016) reported the study of dye-sensitized nanoparticles and their agglomerates with stochastic electrochemistry, and under illumination the cathodic steps are observed because of the photo-oxidation of the dye which injects electrons into the TiO_2_ NP and yields the oxidized dye molecules at particle surface (Barakoti et al., 2016). Peng et al. (2018) developed an ultrasensitive photoelectrochemical method for detecting the photocurrent from single SC nanoparticles by using micrometer-thick nanoparticulate TiO_2_ filmed Au ultramicroelectrode (TiO_2_@AuUME). The presence of di-tetrabutylammonium *cis*-bis(isothiocyanato) bis(2,2′-bipyridyl-4,4′-dicarbox-ylato) ruthenium(II) (N719) makes the TiO_2_ NPs collect more photons and hence increases photoelectrochemical current. They have reported that the electron transfer into the nano-particulate TiO_2_ film will takes place when the individual N719@TiO_2_ nanoparticles stochastically collide with TiO_2_@AuUME (Figure 2A). Coupled with theoretical simulation, the high-resolution photocurrent measurement provides the quantification of electron transfer of single N719@TiO_2_ nanoparticle and the further estimation of the electron diffusivity of TiO_2_@AuUME (Peng et al., 2018). The developed protocol was further used to investigate the photo-electrochemical behavior of single N719@ZnO entity on an Au ultramicroelectrode with different TiO_2_ film thicknesses (Ma et al., 2018). The photocatalytic properties of N719 at single ZnO entity were quantified, and the influence of the film thickness on the electron transport behavior was estimated with the help of simulation. The latter is in agreement with the experimental results, indicating the successful quantification of single SC NPs photoelectrochemistry. Wang et al. demonstrated the light controlled single nanoparticle collision experiments for Pt@TiO_2_ NPs with carbon UME and IrOx NPs with a Nb:TiO_2_ (110) rutile single crystal UME (Figure 2C). The Pt@TiO_2_ NPs was *in-situ* electrochemically deposited and their collisions with a carbon UME as the light guide were catalytically amplified by the oxygen evolution reaction (OER). Current blips due to the collision events was observed. In addition, light-controlled collisions of IrOx NPs with a Nb:TiO_2_ (110) rutile single crystal UME showed photoelectrocatalytic activity of this semiconductor/cocatalyst system. The current spikes can be used to indicate the activities of individual IrO_x_ NPs. Larger current spikes for IrO_x_ NPs than the Pt NPs and Pt@TiO_2_ NPs are associated with the higher electro-catalytical activity of IrO_x_ NPs toward OER (Wang et al., 2020). Overall, the research on single SC photoelectrochemistry are still very limited and only two types of MOS nanoparticles have been investigated. The research objects are highly worthy to be extended so that some general detection strategies could be developed. In addition, more study on the photocurrent behavior of single SC NPs with different properties is required to better understand their intrinsic performance-structure relationships.

## Conclusion

In this mini review the recent research work on the electrochemistry and photoelectrochemistry of single semiconducting nanoparticles are summarized. The redox reactions and electrocatalysis of single metal oxide semiconductor NPs and quantum dots *via* the nano-impact method are discussed. The technique enables both the discoveries of the fundamental physiochemical properties of nanoparticles, and in-depth understanding for the mechanisms and dynamics of (photo) electrochemical processes at single particle levels. However, currently the nano-impact methodology faces some general challenges such as lack of deeper understanding on the dynamics of NPs during the collision process and how different factors have effect on it. For the SC NPs, the substrate-NPs interface plays far more important roles in the electron transfer process due to their semiconducting nature. The influencing factors likely include the electrode materials, surface chemistry of NPs and media composition, which are still poorly understood to date and need to be carefully investigated and clarified in future. Furthermore, the research on single SC NP photoelectrochemistry is still at early stage and the research objects are highly worthy to be extended so that some general detection and analysis strategies could be developed and the intrinsic (photo) electrochemical activity-structure relationships for more SC NPs could be revealed.
